# A comparative analysis of MRI findings versus final pathology in multicentric breast cancer patients at King Abdulaziz Medical City, Jeddah

**DOI:** 10.3389/fsurg.2026.1877150

**Published:** 2026-07-28

**Authors:** Mohammed F. Alotaibi, Abdulrahman S. Alghamdi, Nawaf M. Alharbi, Saleh G. Alqadi, Waleed M. Aljehani, Abdullah M. Balkhi, Jehad S. Alshawi, Muhammad A. Khan

**Affiliations:** 1 Department of General Surgery, King Abdulaziz Medical City, Jeddah, Saudi Arabia; 2 College of Medicine, King Saud bin Abdulaziz University for Health Sciences, Jeddah, Saudi Arabia; 3 King Abdullah International Medical Research Center, Jeddah, Saudi Arabia

**Keywords:** biopsy strategy, breast cancer, final pathology, magnetic resonance imaging, mastectomy, multicentric breast cancer, multifocal breast cancer, neoadjuvant chemotherapy

## Abstract

**Introduction:**

Multicentric breast cancer (MCBC) identified on preoperative breast magnetic resonance imaging (MRI) often influences surgical management toward mastectomy. However, MRI may overestimate true multicentric disease when compared with final pathological findings, potentially leading to overtreatment. This study evaluated the agreement between preoperative MRI and final pathology in diagnosing MCBC at a tertiary oncology center in Jeddah, Saudi Arabia, and explored factors associated with final pathological focality, including biopsy strategy and neoadjuvant chemotherapy (NAC).

**Methodology:**

A retrospective cohort study was conducted at the Princess Noorah Oncology Center, King Abdulaziz Medical City (KAMC), Jeddah, Saudi Arabia, between January 2016 and December 2024. Adult patients (≥18 years) with MRI-diagnosed multicentric breast cancer who subsequently underwent mastectomy were included. Demographic characteristics, tumor and nodal features, risk factors, biopsy findings, treatment modalities, and final pathological focality were retrospectively collected and analyzed.

**Results:**

A total of 233 patients met the inclusion criteria (mean age: 52.76 ± 10.19 years), of whom 102 (43.8%) received NAC. Although all patients were diagnosed with multicentric disease on preoperative MRI, final pathology confirmed multicentric disease in only 25 patients (10.7%). When stratified by NAC status, multicentric disease was confirmed in 4 of 102 patients (3.9%) who received NAC and 21 of 131 patients (16.0%) who did not receive NAC. Among patients who did not receive NAC, biopsy of multiple suspicious lesions was significantly associated with pathologically confirmed multicentric disease (*p* = 0.004), whereas biopsy of a single lesion was more frequently followed by unifocal disease on final pathology.

**Conclusion:**

Preoperative breast MRI demonstrated limited agreement with final pathological findings in patients with presumed multicentric breast cancer, particularly among those who did not receive NAC, in whom only 16.0% had multicentric disease confirmed on final pathology. These findings suggest that histopathological confirmation of all MRI-detected suspicious lesions that may alter surgical management should be considered whenever technically feasible. Integrating MRI with second-look ultrasound, ultrasound-guided biopsy, or MRI-guided biopsy when appropriate may improve diagnostic accuracy, reduce unnecessary mastectomy, and optimize surgical decision-making.

## Introduction

Breast cancer (BC) is a malignant neoplasm arising from the epithelial tissue of the mammary gland and is the most frequently diagnosed cancer among women worldwide. Histologically, breast cancer comprises several subtypes, including invasive ductal carcinoma (IDC), invasive lobular carcinoma (ILC), mixed carcinoma, and other less common variants (1). In addition to histological classification, breast cancer can be categorized according to the distribution of tumor foci within the breast. Unifocal breast cancer is defined as a single discrete tumor within one breast quadrant, whereas multifocal breast cancer refers to the presence of two or more distinct tumor foci within the same breast quadrant, separated by less than 5 cm. In contrast, multicentric breast cancer (MCBC) is characterized by two or more distinct tumor foci located in different breast quadrants or separated by more than 5 cm (2). Breast cancer may also present as unilateral or bilateral disease and can metastasize to regional lymph nodes or distant organs, including the bone, lungs, liver, and brain (3).

Globally, breast cancer remains a major health challenge. It is the most commonly diagnosed cancer among women and one of the leading causes of cancer-related mortality worldwide (4). Approximately one-quarter of all newly diagnosed cancers in women are breast cancers (5). Although advances in screening, diagnosis, and treatment have significantly improved survival, the global burden of breast cancer continues to increase, particularly in low- and middle-income countries where delayed diagnosis and limited healthcare resources contribute to poorer outcomes (5).

In the Kingdom of Saudi Arabia (KSA), breast cancer represents a significant public health concern and is among the most frequently diagnosed malignancies in women (1). Although national screening programs, free access to treatment, and public awareness campaigns have improved early detection and management, the overall burden of the disease continues to increase because of population growth, aging, and increased detection through screening (6). These observations highlight the importance of optimizing diagnostic accuracy and treatment planning to avoid both undertreatment and overtreatment.

The reported incidence of multicentric breast cancer varies considerably, ranging from 6% to 60%, depending on the study population and diagnostic methods used. Data describing the incidence and characteristics of multicentric breast cancer in Saudi Arabia remain limited. Magnetic resonance imaging (MRI) is widely recognized as the most sensitive imaging modality for assessing the extent of breast cancer and detecting additional tumor foci. However, MRI may overestimate disease extent, with lesions appearing multicentric on preoperative imaging later classified as unifocal or multifocal on final pathological examination (7, 8). Consequently, MRI findings may influence surgical decision-making by increasing the likelihood of mastectomy rather than breast-conserving therapy (BCT), particularly in patients with early-stage breast cancer (9).

Accurate assessment of multicentric disease is therefore essential to optimize surgical planning and minimize unnecessary mastectomy while maintaining oncologic safety. Comparing preoperative MRI findings with final pathological results may improve understanding of MRI accuracy and its impact on surgical management. Furthermore, describing the demographic and clinical characteristics of patients with multicentric breast cancer may provide valuable information regarding disease patterns within the Saudi population. Therefore, this study aimed to evaluate the agreement between preoperative breast MRI and final pathological findings in diagnosing multicentric breast cancer at King Abdulaziz Medical City (KAMC), Jeddah, Saudi Arabia.

## Methods

### Study design

This retrospective cohort study was conducted at the Princess Noorah Oncology Center (PNOC), KAMC, Jeddah, Saudi Arabia. The study included patients who underwent preoperative breast MRI and surgical treatment between January 2016 and December 2024. The primary objective was to evaluate the agreement between preoperative MRI findings and final pathological diagnosis in patients with multicentric breast cancer.

### Ethical approval

The study was conducted in accordance with the principles of the Declaration of Helsinki. Ethical approval was obtained from the Institutional Review Board (IRB) of King Abdullah International Medical Research Center (KAIMRC), King Saud bin Abdulaziz University for Health Sciences (KSAU-HS), Jeddah, Saudi Arabia (IRB No. IRB00000139125; Study No. NRJ25/016/2; approved on April 06, 2025).

As this was a retrospective study based on electronic medical records, informed consent was waived by the Institutional Review Board. No patient identifiers were collected, and all data were anonymized before analysis. The data were stored on an encrypted, password-protected computer accessible only to the principal investigator.

### Study population

A total of 388 patients with breast cancer who underwent mastectomy at King Abdulaziz Medical City between January 2016 and December 2024 were identified from the institutional database. Of these, only 233 patients fulfilled the eligibility criteria and were included in the final analysis.

Eligible patients were adults (≥18 years) diagnosed with multicentric breast cancer on preoperative breast MRI who subsequently underwent mastectomy with available final pathological examination. Preoperative MRI was performed before the initiation of neoadjuvant chemotherapy (NAC) in all patients included in the study.

Patients with secondary breast involvement originating from another primary malignancy, those who did not undergo surgical treatment, those without preoperative breast MRI, and those treated with breast-conserving surgery (lumpectomy) were excluded from the study.

### Neoadjuvant chemotherapy

The decision to administer neoadjuvant chemotherapy was made by the institutional multidisciplinary breast tumor board based on the patient's clinical presentation, imaging findings, tumor characteristics, and contemporary treatment guidelines. For all patients who received NAC, the MRI findings analyzed in this study represented the pre-treatment disease burden, as MRI was performed before the initiation of systemic therapy.

### Axillary management

Preoperative axillary lymph node biopsy was performed in patients with clinically or radiologically suspicious axillary lymph nodes. The decision to perform axillary lymph node dissection was based on the results of the preoperative biopsy, intraoperative sentinel lymph node biopsy findings when applicable, and recommendations of the multidisciplinary breast tumor board in accordance with institutional practice.

### Data collection

Data were extracted retrospectively from the electronic medical records using a standardized data collection form. The collected variables included demographic characteristics (age and sex), clinical history (previous malignancy and family history of breast or ovarian cancer), tumor characteristics (histological subtype, tumor stage, MRI findings, and final pathological diagnosis), biopsy findings, preoperative lymph node biopsy results, surgical treatment, axillary management, and risk factors such as smoking status, oral contraceptive use, BRCA1/2 mutations, and Li–Fraumeni syndrome.

### Statistical analysis

Data were entered into Microsoft Excel (Microsoft Corporation, Redmond, WA, USA) and analyzed using IBM SPSS Statistics for Windows (IBM Corp., Armonk, NY, USA). Patients who did not meet the eligibility criteria were excluded before statistical analysis.

Continuous variables were summarized as mean ± standard deviation (SD), whereas categorical variables were presented as frequencies and percentages. Associations between the final pathological diagnosis and categorical variables were evaluated using the Chi-square test or Fisher's exact test, as appropriate. All statistical tests were two-sided, with a *p*-value of <0.05 considered statistically significant. A 95% confidence interval was used for all statistical analyses.

## Results

### Patient characteristics

A total of 233 patients met the study eligibility criteria and were included in the analysis. The mean age was 52.76 ± 10.19 years. A family history of cancer was reported in 43 patients (18.5%), while only 5 patients (2.1%) had a previous history of malignancy. BRCA1 and BRCA2 mutations were identified in three patients (1.3%) each. Fifteen patients (6.4%) were smokers, and 55 (23.6%) reported previous oral contraceptive pill (OCP) use.

NAC was administered to 102 patients (43.8%). Preoperative lymph node biopsy was positive in 110 patients (47.2%), and axillary lymph node dissection was the most frequently performed axillary procedure (135 patients, 57.9%). The most common pathological stage was stage IIB (47 patients, 20.2%).

Although all patients demonstrated multicentric disease on preoperative breast MRI (233/233, 100%), final pathological examination confirmed multicentric disease in only 25 of 233 patients (10.7%). When stratified by neoadjuvant chemotherapy status, multicentric disease was confirmed in 4 of 102 patients (3.9%) who received NAC and 21 of 131 patients (16.0%) who did not receive NAC. All patients underwent mastectomy. The baseline demographic and clinicopathological characteristics are summarized in Table 1.

**Table 1:** Bio-demographic and risk factors.

Variable	Mean	SD
Age	52.76	10.19
	*n* = 233	%
Family history of cancer		
No	190	81.5
Yes	43	18.5
Patient previous cancer history		
No	228	97.9
Yes	5	2.1
BRCA mutation		
No	227	97.4
BRCA1	3	1.3
BRCA2	3	1.3
Li–Fraumeni syndrome		
No	233	100.0
Yes	0	0
Smoking		
No	218	93.6
Yes	15	6.4
OCP		
No	178	76.4
Yes	55	23.6
Neoadjuvant chemotherapy		
No	131	56.2
Yes	102	43.8
MRI report result		
Multicentric	233	100.0
Breast biopsy type for diagnosis		
TruCut	211	90.6
Vacuum assisted	16	6.9
Fine needle	4	1.7
Skin punch	2	.9
Biopsied breast masses		
1 Mass	197	84.5
2 Masses	32	13.7
3 Masses	4	1.7
Presurgical lymph node biopsy		
Not done	81	34.8
Positive	110	47.2
Negative	42	18.0
Postsurgical final pathology report		
Multicentric	25	10.7
Multifocal	94	40.3
Unifocal	114	48.9
Staging (*n* = 226)		
Stage 0	9	4.0
Stage IA	44	19.5
Stage IB	2	.9
Stage IIA	57	25.2
Stage IIB	47	20.8
Stage IIIA	44	19.5
Stage IIIB	6	2.7
Stage IIIC	17	7.5
Treatment for breast cancer		
Mastectomy	233	100.0
Treatment for lymph nodes		
Axillary dissection	135	57.9
Targeted axillary dissection	5	2.1
Sentinel biopsy	93	39.9

### Association between oral contraceptive use and final pathological multicentricity

Among the 25 patients with multicentric disease confirmed on final pathology, 2 patients (8.0%) reported previous OCP use. No statistically significant association was observed between OCP use and multicentricity on final pathological examination (*p* = 0.123) (Table 2).

**Table 2:** OCP correlation with aggressive disease or aggressive axillary treatment.

Variable		OCP				
	Total	No		Yes		*p*
		*n* = 178	%	*n* = 55	%	
Postsurgical final pathology report						
Multicentric	25	23	92.0	2	8.0	0.123^a^
Multifocal	94	72	76.6	22	23.4	
Unifocal	114	83	72.8	31	27.2	
Treatment for lymph nodes						
Axillary dissection	135	102	75.6	33	24.4	0.491^b^
Targeted axillary dissection	5	3	60.0	2	40.0	
Sentinel biopsy	93	73	78.5	20	21.5	

a Chi-squared test.

b Fisher's exact test.

### Association between family history of cancer and final pathological multicentricity

Among patients with multicentric disease confirmed on final pathology, four (16.0%) had a positive family history of cancer. No statistically significant association was identified between family history of cancer and multicentricity on final pathology (*p* = 0.454) (Table 3).

**Table 3:** Family history correlation with aggressive disease.

Variable		Family history of cancer				
	Total	No		Yes		*p*
		*n* = 190	%	*n* = 43	%	
Postsurgical final pathology report						
Multicentric	25	21	84.0	4	16.0	0.454^a^
Multifocal	94	73	77.7	21	22.3	
Unifocal	114	96	84.2	18	15.8	

a Chi-squared test.

### Subgroup analysis of patients who did not receive neoadjuvant chemotherapy

A total of 131 patients did not receive NAC. Within this subgroup, 24 patients (18.3%) had a family history of cancer, 3 (2.3%) had a previous history of malignancy, 23 (17.6%) reported OCP use, and 7 (5.3%) were smokers.

Biopsy was obtained from a single lesion in 106 patients (80.9%). Preoperative lymph node biopsy was positive in 48 patients (36.6%), while axillary lymph node dissection was performed in 72 patients (55.0%). Stage IIA was the most common pathological stage (34 patients, 26.0%).

Although all patients in this subgroup demonstrated multicentric disease on preoperative MRI, final pathology confirmed multicentric disease in 21 patients (16.0%) (Table 4).

**Table 4:** Bio-demographic and risk factors (only no-NAC patients).

Variable	Mean	SD
Age	52.62	10.12
	*n* = 131	%
Family history of cancer		
No	107	81.7
Yes	24	18.3
Patient previous cancer history		
No	128	97.7
Yes	3	2.3
BRCA mutation		
No	127	96.9
BRCA1	3	2.3
BRCA2	1	0.8
Li–Fraumeni syndrome		
No	131	100.0
Yes	0	0
Smoking		
No	124	94.7
Yes	7	5.3
OCP		
No	108	82.4
Yes	23	17.6
Neoadjuvant chemotherapy		
No	131	100.0
Yes	0	0
MRI report result		
Multicentric	131	100.0
Breast biopsy type for diagnosis		
TruCut	115	87.8
Vacuum assisted	13	9.9
Fine needle	1	0.8
Skin punch	2	1.5
Biopsied breast masses		
1 Mass	106	80.9
2 Masses	23	17.6
3 Masses	2	1.5
Presurgical lymph node biopsy		
Not done	61	46.6
Positive	48	36.6
Negative	22	16.8
Postsurgical final pathology report		
Multicentric	21	16.0
Multifocal	52	39.7
Unifocal	58	44.3
Staging (*n* = 126)		
Stage 0	6	4.8
Stage IA	24	19.0
Stage IIA	34	27.0
Stage IIB	25	19.8
Stage IIIA	21	16.7
Stage IIIB	2	1.6
Stage IIIC	14	11.1
Treatment for breast cancer		
Mastectomy	131	100.0
Treatment for lymph nodes		
Axillary dissection	72	55.0
Targeted axillary dissection	1	0.8
Sentinel biopsy	58	44.3

### Association between biopsy strategy and final pathological findings

Among patients who did not receive NAC, a statistically significant association was observed between the number of biopsied lesions and the final pathological diagnosis (*p* = 0.004). Patients who underwent biopsy of a single lesion were more likely to have unifocal disease on final pathology than patients who underwent biopsy of multiple lesions (Table 5).

**Table 5:** Association between clinicopathological characteristics and final pathology in patients who did not receive neoadjuvant chemotherapy (NAC).

Variable		Postsurgical final pathology report						
	Total	Multicentric		Multifocal		Unifocal		*p*
		n	%	n	%	n	%	
Family history of cancer								
No	107	17	15.9	43	40.2	47	43.9	0.971^a^
Yes	24	4	16.7	9	37.5	11	45.8	
Patient previous cancer history								
No	128	20	15.6	51	39.8	57	44.5	0.555^b^
Yes	3	1	33.3	1	33.3	1	33.3	
BRCA mutation								
No	127	21	16.5	49	38.6	57	44.9	0.710^b^
BRCA1	3	0	0.0	2	66.7	1	33.3	
BRCA2	1	0	0.0	1	100.0	0	0.0	
Smoking								
No	124	18	14.5	50	40.3	56	45.2	0.140^b^
Yes	7	3	42.9	2	28.6	2	28.6	
OCP								
No	108	19	17.6	43	39.8	46	42.6	0.514^a^
Yes	23	2	8.7	9	39.1	12	52.2	
Breast biopsy type for diagnosis								
TruCut	115	19	16.5	46	40.0	50	43.5	>0.99^b^
Vacuum assisted	13	2	15.4	5	38.5	6	46.2	
Fine needle	1	0	0.0	0	0.0	1	100.0	
Skin punch	2	0	0.0	1	50.0	1	50.0	
Biopsied breast masses								
1 mass	106	11	10.4	44	41.5	51	48.1	0.004^b^
2 masses	23	9	39.1	7	30.4	7	30.4	
3 masses	2	1	50.0	1	50.0	0	0.0	
Presurgical lymph node biopsy								
Not done	61	10	16.4	23	37.7	28	45.9	0.842^a^
Positive	48	6	12.5	21	43.8	21	43.8	
Negative	22	5	22.7	8	36.4	9	40.9	
Staging								
Stage 0	6	2	33.3	0	0.0	4	66.7	0.447^b^
Stage IA	24	4	16.7	9	37.5	11	45.8	
Stage IIA	34	6	17.6	11	32.4	17	50.0	
Stage IIB	25	3	12.0	14	56.0	8	32.0	
Stage IIIA	21	4	19.0	8	38.1	9	42.9	
Stage IIIB	2	0	0.0	2	100.0	0	0.0	
Stage IIIC	14	1	7.1	7	50.0	6	42.9	
Treatment for lymph nodes								
Axillary dissection	72	11	15.3	32	44.4	29	40.3	0.229^b^
Targeted axillary dissection	1	1	100.0	0	0.0	0	0.0	
Sentinel biopsy	58	9	15.5	20	34.5	29	50.0	

a Chi-squared test.

b Fisher's exact test.

### Association between oral contraceptive use and axillary treatment

Among patients who did not receive NAC, 12 patients (16.7%) who underwent axillary lymph node dissection reported previous OCP use. No statistically significant association was observed between OCP use and axillary treatment (*p* = 0.850) (Table 6).

**Table 6:** OCP correlation with aggressive axillary treatment (only no-NAC patients).

Variable		OCP				
	Total	No		Yes		*p*
		n	%	n	%	
Treatment for lymph nodes						
Axillary dissection	72	60	83.3	12	16.7	0.850^a^
Targeted axillary dissection	1	1	100.0	0	0.0	
Sentinel biopsy	58	47	81.0	11	19.0	

a Fisher's exact test.

### Association between neoadjuvant chemotherapy and final pathological findings

Among the 25 patients with multicentric disease confirmed on final pathology, 21 (84.0%) had not received NAC, whereas 4 (16.0%) had received NAC. Receipt of NAC was significantly associated with a lower proportion of multicentric disease on final pathology (*p* = 0.010) (Table 7).

**Table 7:** Final pathology in NAC versus no NAC.

Variable	Total	Neoadjuvant chemotherapy				
		No		Yes		*p*
		n	%	n	%	
Postsurgical final pathology report						
Multicentric	25	21	84.0	4	16.0	0.010^a^
Multifocal	94	52	55.3	42	44.7	
Unifocal	114	58	50.9	56	49.1	

a Chi-squared test.

## Discussion

The present study evaluated the agreement between preoperative breast MRI and final pathological findings in patients diagnosed with MCBC at a tertiary care center in Saudi Arabia. The principal finding was the substantial discrepancy between MRI-diagnosed multicentric disease and the final pathological diagnosis. Although all included patients were diagnosed with multicentric disease on preoperative MRI, among patients who did not receive NAC, multicentric disease was confirmed in only 16.0% of cases on final pathology. Because MRI was performed before systemic therapy, this subgroup more accurately reflects the diagnostic performance of MRI. In contrast, the substantially lower rate observed in patients who received NAC (3.9%) is likely attributable to treatment response rather than reduced MRI specificity. In addition, biopsy of multiple suspicious lesions was associated with a significantly higher rate of pathologically confirmed multicentric disease compared with biopsy of a single lesion. No significant associations were observed between OCP use or family history of cancer and pathological multicentricity.

### Patient characteristics

The demographic and clinical characteristics of our cohort were generally consistent with those reported in previous studies. The mean age of the study population was comparable to that reported by Basudan (1) and Omer et al. (4), supporting the observation that breast cancer in Saudi Arabia is commonly diagnosed in women in their early 50s.

Most patients did not report a family history of cancer or a previous history of malignancy, consistent with the established understanding that the majority of breast cancers occur sporadically, with only approximately 5%–10% attributable to hereditary syndromes. Similarly, BRCA1 and BRCA2 mutations were identified in only a small proportion of patients, which is consistent with previous studies reporting low mutation frequencies among unselected breast cancer populations (10–12).

Nearly half of the patients had positive preoperative axillary lymph node biopsy findings, and axillary lymph node dissection was the most frequently performed axillary procedure. This management strategy reflects current surgical practice, in which patients with biopsy-proven nodal metastases generally undergo axillary lymph node dissection, whereas sentinel lymph node biopsy is reserved for patients without clinically evident nodal disease or with limited nodal involvement, in accordance with contemporary guidelines (13).

### MRI–pathology discrepancy

The most important finding of this study was the marked discrepancy between MRI findings and final pathological examination. Despite all patients demonstrating multicentric disease on preoperative MRI, only a small proportion were confirmed to have true multicentric disease after surgery. Similar observations have been reported in previous studies, demonstrating that, although breast MRI has excellent sensitivity for detecting additional tumor foci, its specificity is variable and may result in overestimation of disease extent (14–16).

Several factors may explain this discrepancy. MRI frequently detects additional enhancing lesions that may represent benign proliferative changes, background parenchymal enhancement, or non-malignant abnormalities rather than true malignant tumor foci. Consequently, MRI findings alone should not be considered definitive evidence of multicentric disease when major surgical decisions, such as mastectomy, are being considered.

Importantly, interpretation of these findings should consider that MRI was performed before the initiation of NAC, whereas final pathology was obtained after surgical treatment. Therefore, among patients who received NAC, treatment response may have reduced the pathological extent of disease, thereby contributing to the observed discrepancy between imaging and pathology.

Current evidence supports a multimodality approach when MRI identifies additional suspicious lesions. Second-look ultrasound, targeted ultrasound-guided biopsy, and MRI-guided biopsy, when appropriate, may improve diagnostic accuracy and reduce unnecessary mastectomy by confirming whether additional MRI-detected lesions represent true malignant disease (17–19).

### Impact of biopsy strategy

The number of biopsied lesions was the only variable significantly associated with final pathological multicentricity. Patients who underwent biopsy of multiple suspicious lesions were substantially more likely to have multicentric disease confirmed on final pathology than those who underwent biopsy of only a single lesion.

These findings suggest that limited tissue sampling may contribute to discrepancies between preoperative imaging and final pathology. However, this association should not be interpreted as evidence that biopsy itself alters tumor focality; rather, biopsy of additional suspicious lesions may improve preoperative characterization of disease extent when the results are expected to influence surgical management.

Based on our findings, we propose that all MRI-detected suspicious lesions suspected of representing multicentric breast cancer should undergo histopathological confirmation before proceeding to mastectomy whenever technically feasible. Although this approach may require additional biopsies, it has the potential to improve diagnostic accuracy, reduce unnecessary mastectomy resulting from MRI overestimation of disease extent, and optimize surgical decision-making

### Oral contraceptive Use and family history

Neither previous OCP use nor family history of cancer demonstrated a significant association with pathological multicentricity or the extent of axillary surgery. These findings suggest that, although OCP use and family history are recognized risk factors for the development of breast cancer, they do not appear to predict disease focality or surgical aggressiveness in patients with established breast cancer. Previous studies have similarly demonstrated inconsistent associations between OCP exposure and tumor biology, with stronger evidence linking OCP use to breast cancer incidence rather than disease aggressiveness (20, 21).

### Effect of neoadjuvant chemotherapy

Patients who received NAC were significantly less likely to have multicentric disease confirmed on final pathology than patients who did not receive NAC. Because MRI was performed before systemic therapy in all patients, this finding most likely reflects tumor response to chemotherapy rather than reduced diagnostic performance of MRI. Previous studies have shown that NAC may substantially reduce tumor burden and, in some patients, achieve pathological complete response, supporting this interpretation (22).

### Limitations

This study has several limitations. First, its retrospective single-center design may limit the generalizability of the findings. Second, the sample size was relatively small compared with previously published studies. Third, only patients with MRI-diagnosed multicentric breast cancer who underwent mastectomy were included, introducing the possibility of selection bias. Fourth, although preoperative MRI was performed before the initiation of NAC, pathological assessment was conducted after treatment in patients who received NAC, which may have influenced the observed discrepancy between MRI findings and final pathology. Finally, the absence of a universally accepted definition of multicentric breast cancer may have affected case classification and limited comparability with other studies.

## Conclusion

Preoperative breast MRI demonstrated a substantial discrepancy with final pathological findings in the assessment of multicentric breast cancer. Although all patients were diagnosed with multicentric disease on preoperative MRI, among patients who did not receive neoadjuvant chemotherapy, only 21 of 131 patients (16.0%) had multicentric disease confirmed on final pathology, suggesting that MRI may overestimate disease extent in this setting. Among patients who did not receive neoadjuvant chemotherapy, biopsy of multiple suspicious lesions was associated with a significantly higher rate of pathologically confirmed multicentric disease than biopsy of a single lesion, suggesting that more comprehensive tissue sampling may improve preoperative diagnostic accuracy.

Based on these findings, we recommend histopathological confirmation of all MRI-detected suspicious lesions that are expected to influence surgical management before proceeding to mastectomy for presumed multicentric breast cancer, whenever technically feasible. This may be achieved using second-look ultrasound with ultrasound-guided biopsy or MRI-guided biopsy when appropriate. Such an approach has the potential to improve diagnostic accuracy, reduce unnecessary mastectomy, and optimize surgical decision-making. Further prospective, multicenter studies are warranted to validate these findings and establish standardized diagnostic pathways for patients with suspected multicentric breast cancer.

## Data Availability

The original contributions presented in the study are included in the article/Supplementary Material; further inquiries can be directed to the corresponding author/s.
